# Hip Involvement in Pediatric Scurvy: Early Magnetic Imaging Signs

**DOI:** 10.3390/children12050642

**Published:** 2025-05-16

**Authors:** Lisa Gamalero, Anna Perrone, Chiara Macucci, Alessandra Meneghel, Marta Balzarin, Sandra Trapani, Giuseppe Indolfi, Giorgia Martini, Teresa Giani

**Affiliations:** 1 Paediatric Division, ULSS2 Marca Trevigiana, Conegliano Hospital, 31015 Conegliano, Italy; 2 Radiology Unit, AOU Meyer IRCCS, 50139 Florence, Italy; 3 Department of Pediatrics, AOU Meyer IRCCS, 50139 Florence, Italy; 4 Department of Woman’s and Child’s Health, University of Padua, 35122 Padua, Italy; 5 Pediatric Unit, “San Bortolo” Hospital, 36100 Vicenza, Italy; 6 Paediatric Division, Academic Hospital of Udine, 33100 Udine, Italy

**Keywords:** hip, juvenile idiopathic arthritis, magnetic resonance imaging, sacroiliac joints, scurvy, vitamin C

## Abstract

**Background:** Scurvy is an uncommon and often underrecognized disease. However, conditions associated with a restrictive and/or selective diet and inadequate absorption still pose a high risk for developing vitamin C deficiency. Musculoskeletal symptoms are among the most characteristic manifestations of scurvy, often requiring radiological investigations. **Objective:** This study aims to describe the radiological signs of scurvy on pelvic magnetic resonance imaging (MRI) in children presenting with musculoskeletal symptoms and to highlight features that may help differentiate it from other conditions with similar presentations. **Methods:** We conducted a retrospective study including children admitted for musculoskeletal symptoms requiring a pelvic MRI and who were subsequently diagnosed with scurvy. Demographic, clinical, laboratory, and radiological data were extracted from electronic medical records. **Results:** We identified ten patients with a median age at disease onset of 45 months (range 17–133 months) admitted between 2016 and 2022. All ten patients included in the study were male. All had at least one of the following symptoms: limping, pain in the lower limbs, or refusal to walk, in addition to gum bleeding (7/10), hypertrophic gums (5/10), purpura (3/10), irritability (3/10), and fever (2/10). In all patients, pelvic MRI showed a bilateral, patchy, abnormal, water-like signal intensity pattern in the sacroiliac area. Sacroiliitis was detected in three children and hip effusion in another child. Seven out of these ten patients had a previous pelvis X-ray that was negative. **Conclusions:** In scurvy, the pelvis is often prematurely affected, with bone marrow accumulating water and joints showing inflammatory changes, particularly at the hips and sacroiliac joints. Due to its ability to assess soft tissues and its high sensitivity to water content, MRI is the ideal imaging tool to assess these changes. In contrast, plain radiography is less sensitive and specific and may be uninformative in the early stages of the disease.

## 1. Introduction

Scurvy is an ancient disease caused by a chronic vitamin C deficiency, typically resulting from poor dietary intake of fresh fruit and vegetables or malabsorption [1,2]. Although considered rare, scurvy has seen a resurgence in recent years, even in high-income countries, likely driven by socio-economic challenges such as rising food insecurity and limited access to fresh produce, as well as by increased clinical awareness and recognition of nutritional deficiencies in specific pediatric populations. Children with restrictive eating behaviors, such as anorexia nervosa, or underlying conditions impairing nutrition, including autism spectrum disorders, malabsorption syndromes, or chronic illnesses, represent high-risk groups [3,4].

Scurvy is a preventable and treatable condition; however, it often remains underdiagnosed due to its non-specific early symptoms and clinical overlap with more common pediatric diseases. The lack of systematic epidemiological data and limited clinical awareness further contribute to diagnostic delays, highlighting the need for heightened suspicion among healthcare providers.

Vitamin C deficiency is defined by serum values less than 0.75 mg/dL, with symptomatic children often presenting levels below 0.25 mg/dL [5]. Clinical features typically appear one to three months after a significant reduction in the intake or absorption of vitamin C and are usually characterized by irritability and anorexia [1,2]. Mucocutaneous manifestations, such as poor wound healing, gingival swelling and bleeding, tooth loss, petechiae, ecchymoses, hyperkeratosis, and subconjunctival hemorrhages, eventually become evident [6]. Musculoskeletal symptoms such as arthralgia, myalgia, back pain, weakness, inability to bear weight, and limping often prompt medical attention. However, the involvement of the hip, particularly in its early stages, has been poorly characterized in the literature and may mimic other diseases, leading to misdiagnosis and diagnostic delays.

Hemarthrosis, subperiosteal hemorrhage, and fractures due to compromised endochondral bone formation may also occur. In advanced stages, the disease can be lethal, predisposing to anasarca, hemolysis, jaundice, and convulsions [6,7]. Treatment involves supplementation with oral or intravenous ascorbic acid [8].

The phenotypic heterogeneity of scurvy poses several challenges, as its clinical manifestations reflect the widespread role of vitamin C in connective tissue integrity, hematopoiesis, immune function, and collagen synthesis. The differential diagnosis is broad and includes rheumatological, orthopedic, neurological, and hematological diseases [9]. Conditions such as juvenile idiopathic arthritis (JIA), septic arthritis, malignancies, and severe malnutrition-related disorders may share clinical features, including musculoskeletal pain, weakness, fatigue, fever, mucocutaneous bleeding, inability to bear weight, and elevated inflammatory markers. The diagnosis of scurvy is often delayed, with a median interval of two months (and up to years) from the onset of clinical symptoms [1].

Imaging plays a crucial role in the initial diagnostic process. While plain radiography is fast and cost-effective, magnetic resonance imaging (MRI) offers greater sensitivity in detecting early bone lesions and provides a more detailed evaluation of soft tissues, and it is increasingly employed in the assessment of children presenting with limping or refusal to walk.

Radiological studies focusing specifically on MRI features of pediatric scurvy are scarce, and systematic descriptions of pelvic findings are largely lacking. The pelvis represents an ideal anatomical site for detecting early changes, due to its abundant bone marrow content and mechanical stress load. Moreover, MRI can detect subtle changes in tissue’s water content and initial marrow and soft tissue alterations, which are not yet visible on conventional radiographs, potentially aiding earlier recognition of scurvy before more severe damage occurs.

Although scurvy remains an unusual cause of musculoskeletal symptoms, its clinical overlap with other disorders underscores the importance of assessing dietary habits and considering vitamin C deficiency in differential diagnosis. In addition, defining the characteristic pelvic MRI findings of scurvy may aid in early recognition and facilitate appropriate management.

We aim to describe the radiological signs of vitamin C deficiency on pelvic MRI in children with scurvy presenting with musculoskeletal symptoms. Additionally, we seek to identify imaging features that can help differentiate scurvy from other conditions with similar presentations.

## 2. Material and Methods

### 2.1. Data Collection

We analyzed data from patients admitted for musculoskeletal manifestations between 2016 and 2022 who were subsequently discharged with a diagnosis of scurvy based on the International Classification of Diseases (ICD). Only patients who had undergone pelvic MRI were included in the study.

The diagnosis of scurvy was based on a combination of compatible clinical features and the exclusion of alternative diagnoses and confirmed by low serum vitamin C concentrations (normal range: 460–1400 mcg/dL).

The data were extracted from electronic medical records and imaging archives. Available data from other radiological investigations performed within one month prior to pelvic MRI were also collected when present. All extracted data were independently verified by a second investigator to ensure accuracy.

### 2.2. MRI Image Evaluation

Pelvic MRI scans, as well as lower limb MRIs when available, were performed before and after intravenous gadolinium administration. All images were reviewed by a single experienced pediatric radiologist. In cases of uncertainty, findings were discussed with a second senior radiologist until a consensus was reached. All imaging abnormalities were systematically described, with the primary aim of characterizing the spectrum of MRI findings observed in this pediatric cohort with a confirmed diagnosis of scurvy.

The evaluation focused on the presence, pattern, and distribution of bone marrow signal abnormalities in the pelvic bones, classified as either patchy (referring to non-uniform, heterogeneous signal abnormalities reflecting variations in water content), or homogeneous, and recorded as unilateral or bilateral.

Sacroiliac joint involvement was assessed by evaluating the presence or absence of post-contrast enhancement and/or joint effusion, again specifying laterality. Additional musculoskeletal findings, such as hip joint effusion, signal changes in the proximal femora, periosteal reactions, signal abnormalities of the lumbar vertebrae, and edema of adjacent soft tissues, were also recorded and their distribution described when applicable.

### 2.3. MRI Protocol

MRI examinations were performed using 1.5 tesla (T) or 3T systems (Achieva; Achieva; Philips Medical Systems, Best, The Netherlands). The MRI protocol included coronal or axial T1-weighted images (T1WI) to evaluate anatomical details; coronal and axial fluid-sensitive sequences, such as short tau inversion recovery (STIR), and T2-weighted fat-saturated images (T2FS), to detect edema and inflammation by suppressing fat signals; axial T1-weighted fat-saturated sequences, both pre and post contrast-enhanced, to assess enhancement patterns after gadolinium-based contrast administration; and a dynamic contrast-enhanced coronal T1-weighted sequence acquired using T1 high-resolution isotropic volume examination (THRIVE) or Dixon techniques, providing detailed information on tissue vascularization and dynamic enhancement patterns. Additionally, T1 turbo spin echo (TSE) sequences without fat saturation were included to detect subtle changes in bone marrow composition, as they are highly sensitive to alterations in marrow signal.

### 2.4. Ethical Considerations

This retrospective study used anonymized data, in line with institutional policies and applicable regulations, with parental consent obtained for the use of clinical data for research purposes. The study was conducted in accordance with the Declaration of Helsinki.

## 3. Results

We identified 14 pediatric patients diagnosed with scurvy during the study period. Four patients were excluded as they did not undergo pelvic MRI. Therefore, ten patients were included in the analysis. All patients were male.

The median age at disease onset was 45 months (range 17–133 months). The median diagnostic delay was 2 months (range 1–33 months).

At presentation, the most common symptoms were limping (8/10, 80%), gum bleeding (7/10, 70%), hypertrophic gums (5/10, 50%), refusal to walk (4/10, 40%), skin bleeding (3/10, 30%), lower limb pain (3/10, 30%), back pain (3/10, 30%), irritability (3/10, 30%), thigh swelling (2/10, 20%), fever (2/10, 20%), nocturnal awakening due to lower limb pain (1/10, 10%), and tooth pain (1/10, 10%).

Over time, all patients developed musculoskeletal manifestations, including limping, lower limb pain, and refusal to walk.

One patient had a severe disease course, initially characterized by left leg pain, refusal to walk, and thigh swelling, which rapidly progressed to hypovolemic shock.

Eight out of ten patients had a concomitant condition: 3/10 (30%) had autism spectrum disorder, 2/10 (20%) had epilepsy and developmental delay, 1/10 (10%) had glucose-6-phosphate deficiency (G6PD) and developmental delay, 1/10 (10%) had language delay, and 1/10 (10%) had multiple food allergies. All patients adhered to a highly selective diet with minimal or no intake of fruits and vegetables. Vitamin C levels were measured in all patients, showing severe deficiency in all children. The main clinical and laboratory data, along with the initial diagnostic suspicions, are summarized in Table 1.

All patients underwent gadolinium-enhanced MRI of the pelvis and lower limbs, and one patient also underwent whole-body MRI (WB-MRI). In all cases, MRI was performed due to musculoskeletal pain associated with limping or refusal to ambulate, aiming to better define the affected areas, and their localization, and to support the differential diagnosis process. The MRI findings were systematically analyzed according to the anatomical regions involved: sacroiliac joints, hip joints, and bone marrow. Pelvic MRI showed bilateral involvement in all patients (10/10, 100%). A patchy distribution of bone marrow signal alteration was observed in 9 out of 10 patients (90%), while a homogeneous signal alteration was identified in 1 patient (10%). Gadolinium-enhanced imaging showed sacroiliac joint enhancement in 8/10 patients (80%), while 2/10 (20%) showed no post-contrast enhancement at this level. Additional MRI findings beyond sacroiliac and pelvic bone marrow involvement included hip joint effusion, signal changes in the proximal femora, periosteal reaction, signal abnormalities of the lumbar vertebrae, and edema of adjacent soft tissues, as detailed in Table 2.

Eight children also underwent X-rays prior to MRI, with a median time interval of 9 days (range 3–27 days). Seven of them underwent pelvic X-rays and three underwent lower limb X-rays. Among these, two showed non-specific findings, while one displayed radiographic signs consistent with scurvy. Among these, two showed non-specific findings, while one displayed radiographic signs consistent with scurvy (Table 2).

All patients received oral vitamin C supplementation, with a subsequent rapid resolution of symptoms. One patient underwent a follow-up WB-MRI three months later, which showed normalization. Four patients (40%) underwent a follow-up plain radiograph of the lower limbs; in one patient, a mild alteration persisted after five months, while in two others, diffuse osteopenia remained evident after two and six months of vitamin C supplementation, respectively. In the fourth patient, a plain radiograph of the lower limbs was performed for the first time only after five months of supplementation and was negative.

## 4. Discussion

Vitamin C exerts deep and pleiotropic effects on the skeletal system. It acts as the cofactor of prolyl and lysyl hydroxylases, essential enzymes for collagen cross-linking, which ensure the formation of a well-organized collagen network, especially in bones and vessel walls [10,11]. Collagen is a triple-helical protein essential for connective tissue strength. Its stability depends on the hydroxylation of specific proline residues, which, due to their ability to attract electrons, promote proper helix folding. Vitamin C is a cofactor, playing a crucial role in enabling proline hydroxylation, making it essential for collagen stability. In its absence, collagen fibers are poorly formed and inadequately cross-linked, leading to the increased fragility of blood vessels, impaired osteoid matrix formation, and defective connective tissue integrity. Additionally, ascorbic acid neutralizes free radicals and toxins, promotes iron absorption, stabilizes folic acid and vitamin E, exerts multi-directional influences on osteoblasts and osteoclasts, regulates gene expression, and mitigates inflammation [12,13,14].

Ascorbate deficiency compromises endochondral ossification, leading to bone resorption in both trabecular and cortical bone tissues [11,13]. This process predisposes to bone pain, limping, and bleeding, which may occur in mucocutaneous, intra-articular, or sub-periosteal locations [10,11].

Skeletal lesions typically affect areas with significant growth and high collagen synthesis, including the distal femur, proximal tibia, proximal fibula, distal radius, distal ulna, and proximal humerus [15,16,17,18]. This highlights how musculoskeletal symptoms are frequently expressed in scurvy, even though the disease itself remains an uncommon cause of bone pain and unexplained limping. Nevertheless, scurvy still occurs in high-income countries, particularly among pediatric populations with restrictive diets, neurodevelopmental disorders, or chronic illnesses. Therefore, scurvy should be included in the differential diagnosis of musculoskeletal pain in children, particularly among high-risk groups, and a careful assessment of nutritional history should be performed in any child presenting with unexplained musculoskeletal symptoms, such as a limp.

In our cohort of ten pediatric patients, the median age at disease onset was 3 years and 8 months. Most children had underlying conditions associated with restrictive eating behaviors or neurodevelopmental disorders. Limping was the predominant symptom, often accompanied by features such as bleeding, hypertrophic gums, and fever. Elevated inflammatory markers and anemia were commonly detected, further complicating the initial diagnostic process, with early diagnostic suspicions mainly directed towards inflammatory or oncologic conditions rather than nutritional deficiencies. The median diagnostic delay was 2 months. Definitive confirmation of scurvy was obtained through markedly reduced plasma vitamin C levels in all patients tested.

Pelvic MRI was performed at a median of 2 months after symptom onset. Bone marrow bilateral signal abnormalities in the pelvis were identified in all patients and were often associated with inflammation-like signs of sacroiliac joint involvement. In most cases, previous conventional X-rays of the pelvis or hips were either normal or showed only non-specific findings.

**Figure 1:**
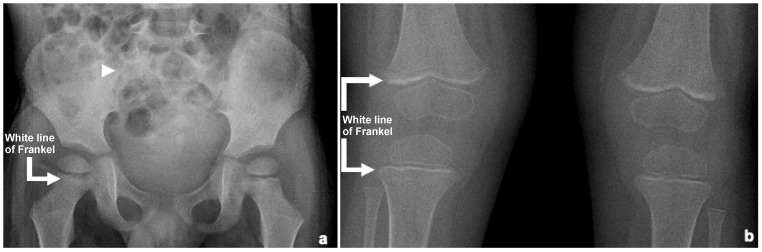
Radiological images from a three-year-and-nine-month-old boy with scurvy. (**a**) An anteroposterior hip radiograph shows no abnormalities in the sacroiliac joints (arrowhead). A mild dense band is observed in the metaphysis of the proximal femur (arrow). Superim position of bowel meteorism is also noted; (**b**) An anteroposterior knee radiograph reveals increased density in the metaphyses of the distal femur and proximal tibia bilaterally (arrows). A diffuse reduction in bone mineral density is also present.

Early findings on conventional X-ray may be absent or subtle and non-specific, such as a diffuse pattern of demineralization, while classic signs typically appear three to six months later [1]. Figure 1 shows nonspecific radiographic findings on knee and pelvic X-rays.

Although the role of MRI in scurvy has been minimally described, this imaging modality has proven to be both sensitive and accurate in assessing the musculoskeletal structures that are frequently affected in scurvy [15,18,19]. MRI can detect cortical thinning, periosteal edema, and mineral deposition secondary to subperiosteal hemorrhage. Furthermore, it provides a comprehensive visualization of bone marrow subversion, which occurs in conditions characterized by severe malnutrition [20]. Bone marrow is a semi-solid soft tissue primarily located within the central cavity of long bones and the spaces of cancellous bones, such as the ribs, vertebrae, sternum, and bones of the pelvis. In scurvy, the fat cells that are crucial for maintaining hematopoietic progenitor cells tend to disappear and be replaced by a hyaluronic acid-like material, attracting a large amount of water. This alteration predisposes to the so-called gelatinous bone marrow subversion [20]. This process adversely affects the hematopoietic microenvironment, compromising bone marrow’s structural integrity and leading to hematopoietic tissue hypoplasia [20]. The underlying pathophysiological mechanisms remain unclear; however, conditions of significant stress, such as malnutrition, are thought to impact bioregulatory processes, potentially disrupting the balance of hematopoietic and stromal cell interactions.

**Figure 2:**
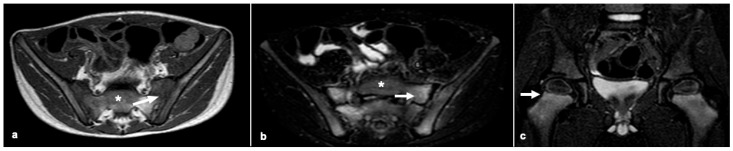
Magnetic resonance images in a four-year-old boy with scurvy. (**a**) An axial pcst-contrast T1 image shows bilateral, symmetric multifocal water-like signals in the ilium and sacrum (arrow); (**b**) An axial short tau inversion recovery (STIR) demonstrates sacroiliac contest-enhancement (arrow). In panels (**a**,**b**), (*) indicates bone marrow with normal appearance in the sacral region. (**c**) A coronal STIR image showing bilateral hyperintensities in the proximal femoral metaphysis (arrow).

In the early stages of malnutrition, changes are focal but become patchy and eventually diffuse with a bilateral and symmetric distribution as the condition persists [21,22,23,24,25]. The pathophysiological consequences of vitamin C deficiency directly translate into imaging abnormalities. Due to its high sensitivity to subtle changes in water content, MRI is the ideal imaging modality for identifying these evocative, albeit non-specific, signs [22]. Additionally, bone marrow, with its inherent soft tissue contrast and non-ionizing nature, is particularly well suited for evaluation by MRI, as shown in Figure 2 [26].

In our cohort, a bilateral and patchy distribution of abnormal water-like MRI signals was detected in the hips of all children. We hypothesize that these radiological pelvic findings are related to the high bone marrow content in this region and its gelatinous transformation associated with scurvy.

Water-sensitive sequences such as fat-suppressed proton-density-weighted, fat-suppressed T2-weighted, and/or short tau inversion recovery sequences are the most suitable for detecting these bone marrow changes.

This bilateral and symmetrical distribution of bone marrow signal abnormalities may assist in differential diagnosis by helping to distinguish scurvy from malignant conditions such as leukemia, lymphoma, or metastatic disease, which more commonly present with focal and asymmetric patterns of involvement. Pelvic Langerhans cell histiocytosis, by contrast, typically presents with well-demarcated, focal lytic lesions that are often asymmetric. Another condition that may mimic scurvy on pelvic MRI is the presence of red marrow islands. These are areas of normal hematopoietic marrow, commonly seen in children, particularly in the metaphyseal and pelvic regions. Compared to scurvy-related abnormalities, red marrow islands are typically located subcortically or subchondrally, and often exhibit a characteristic flame-shaped appearance. On STIR/T2-weighted sequences, they show a mild and diffuse hyperintensity, whereas signal changes associated with scurvy tend to be more confluent and of higher intensity.

Beyond marrow signal abnormalities, we identified additional pelvic MRI findings that closely resembled those typically seen in inflammatory bone and joint conditions, raising the potential for misdiagnosis. We observed increased gadolinium enhancement at the sacroiliac joints in three patients; in one case, this was accompanied by joint effusion. Hip joint effusion was also detected in two children, unilateral in one case and bilateral in the other. However, contrary to initial impressions, these findings are more likely the radiological counterpart of the collagen-related molecular alterations induced by ascorbic acid deficiency, reflecting impaired collagen synthesis and compromised connective tissue integrity. These radiological signs seem to reflect the increased vascular permeability and reparative activity around the periosteum and bone, often secondary to subperiosteal hemorrhage. Additionally, irritation caused by adjacent periosteal or subperiosteal involvement, such as microfractures, edema, or bleeding, could account for the presence of joint effusion. Moreover, the imbalance between reduced osteoblast activity and increased osteoclast proliferation, leading to disrupted bone homeostasis, may further contribute to MRI inflammation-like findings.

Considering these aspects, the early involvement of the pelvis in pediatric scurvy may be explained by the high proportion of active hematopoietic marrow in the pelvic bones, and its vulnerability to microvascular fragility and impaired collagen synthesis. Moreover, the mechanical loading of the hip region could further exacerbate the manifestation of early symptoms and imaging findings. However, the literature provides limited data on sacroiliac involvement in scurvy, primarily from single-case reports [18,27,28]. A recent literature review summarized findings from 166 children across 15 studies; in total, 38% presented with refusal to walk, 16% with limping, and 0.6% with back pain [1]. Thirteen of these patients underwent sacroiliac MRI, which was abnormal in nine cases (69.2%); six of them had previously negative pelvis X-rays. Abnormal MRI findings included bone marrow signal alterations in five patients, contrast enhancement in two, soft tissue involvement in four, and sacroiliac joint effusion in two. Additional findings included multifocal bone marrow signal abnormalities in the metaphyses of lower limbs in one patient, in the upper limbs in another, and intra-medullary infiltration of multiple vertebrae in a third patient.

Radiological signs of sacroiliitis and coxitis, combined with musculoskeletal symptoms and increased inflammatory markers, may prompt consideration of a differential diagnosis with JIA. Among all JIA subtypes, enthesitis-related arthritis (ERA) warrants particular attention, as it most closely mimics scurvy due to its predominant involvement of the sacroiliac and coxofemoral joints [18,29,30].

In ERA, sacroiliac MRI is the gold-standard imaging modality for identifying early active inflammation before the onset of structural changes. However, no validated MRI-based scoring systems for sacroiliitis exist for pediatric patients, and specific radiological findings are lacking [31]. In this condition, edema and synovial enhancement are the most common initial signs of inflammation, and radiological features are often asymmetric and focal, with an intense high signal on fat-suppressed T2-weighted images [32]. Table 3 highlights key signs that may assist in the differential diagnosis of pelvic MRI findings in scurvy versus JIA/ERA.

Given the systemic nature of scurvy, which can present with multiple and diffuse lesions, whole-body MRI (WB-MRI) may represent a more suitable imaging modality compared to targeted MRI. WB-MRI allows for a comprehensive assessment of systemic involvement in such conditions [33]. Despite its potential benefits, only a single case report has documented the use of WB-MRI in this disease [34]. In our series, one patient underwent WB-MRI, which enabled early diagnosis through the detection of various, widespread, typical musculoskeletal lesions. Additionally, WB-MRI is valuable for differential diagnosis, as radiological signs of inflammation in the peripheral joints, axial skeleton, and enthesis can support the diagnosis of ERA [35]. Although data are lacking, whole-body MRI could be considered in patients with widespread musculoskeletal symptoms, atypical clinical presentations, inconclusive findings on targeted imaging, or when a systemic disease, such as scurvy, is suspected from the outset. In these cases, the identification of multiple lesions, their appearance, and their distribution may significantly assist in guiding the diagnosis.

This study has several limitations. First, the retrospective design and small sample size limit the generalizability of the findings. Moreover, as MRI was not performed in all cases of pediatric scurvy during the study period, there may be a selection bias toward more severe or atypical presentations. Lastly, while the MRI findings were systematically reviewed, the absence of a control group or comparison with other diagnostic modalities, such as ultrasound or CT, limits the ability to assess the specificity of the described imaging patterns.

Studies involving larger cohorts are needed to confirm the MRI findings described in our cohort and to establish standardized imaging protocols for the early diagnosis of pediatric scurvy. Early recognition through MRI could help expedite diagnosis and treatment, while avoiding unnecessary investigations or inappropriate therapies.

## 5. Conclusions

In clinical practice, pelvic MRI can be considered in children presenting with unexplained musculoskeletal symptoms, especially in the presence of risk factors for nutritional deficiencies. Pelvic involvement in children occurs early and may mimic other musculoskeletal conditions. MRI is more sensitive than plain radiography in detecting early bone marrow and joint changes, including bone marrow gelatinous transformation and joint effusion, which are well documented in the pelvic region.

Given the systemic nature of scurvy, WB-MRI may represent a valuable tool in the diagnostic work-up of suspected hypovitaminosis C.

Prompt scurvy recognition can prevent misdiagnosis and ensure the timely treatment of this rare but reversible condition.

Further prospective studies are needed to validate these MRI findings in larger cohorts and to compare the diagnostic utility of WB-MRI with targeted MRI.

## Figures and Tables

**Table 1:** Main clinical and laboratory data of the patients described.

Patient N	Age at Disease Onset (Months)	Diagnostic Delay (Months)	Associated Disease	Signs/Symptomps of Scurvy						Initial Diagnostic Suspicion	Elevated CRP/ESR	Anemia	Plasma Vitamin C Level
				Limping	Back Pain	Bleeding	Hypertrophic Gums	Fever	Other				
1	48	2	Autism, ED	YES	YES	YES	YES	NO	Bilateral thigh swelling	Spondylodiscitis, neoplastic disease	Both	YES	36 mcg/dL
2	29	6	Multiple food allergies (milk, egg, fish)	YES	NO	YES	YES	NO	Thigh swelling, hypovolemic shock, polyserositis, irritability	Osteomyelitis, myosarcoma	CRP	YES	3 mcg/dL
3	17	3	None	NO	NO	YES	YES	N	NO	Transient hip arthritis, osteomyelitis, CRMO, JIA	Both	YES	26 mcg/dL
4	133	1	Severe developmental delay, spastic tetraparesis, focal epilepsy	NO	NO	YES	YES	YES	Tooth pain, refusal to walk, nocturnal lower limb pain	Osteomyelitis, muscle contracture	Both	YES	Undetectable
5	45	1	None	YES	NO	YES	NO	NO	Right thigh pain, refusal to walk, irritability	Transient hip arthritis, osteomyelitis, CRMO	NO	YES	53 mcg/dL
6	43	1	Autism, ED	YES	NO	NO	NO	NO	NO	Spondylodiscitis	ESR	YES	Undetectable
7	120	2	Rett syndrome	YES	NO	YES	NO	YES	Right leg pain, swelling, and irritability	Osteomyelitis	Both	YES	Undetectable
8	45	2	Autism	YES	YES	NO	NO	NO	NO	JIA (ERA subtype)	NO	Unknown	Undetectable
9	38	1	Psycomotor delay, G6PD, drepanocytotosis	YES	YES	NO	NO	NO	NO	JIA (ERA subtype)	NO	YES	Undetectable
10	44	33	Language delay	YES	NO	YES	YES	NO	NO	JIA	Both	YES	Undetectable

CRMO, chronic recurrent multifocal osteomyelitis; G6PD, glucose-6-phosphate dehydrogenase; CRP, C-reactive protein; ESR, erythrocyte.

**Table 2:** Summary of the main imaging studies performed in the described patients.

Patient N	Interval Between Symptoms and MRI (Months)	MRI: Whole Body/Targeted	Pelvic Involvement: Bilateral/Monolateral	Pelvic Marrow Signal Pattern	Sacroiliac Gadolinium Enhancement	Additional Radiological Signs	Previous X-Ray: Anatomical Area and Result
1	2	Whole Body	Bilateral	Patchy	Present	Edema of adjacent soft tissues and symmetrical signal changes in vertebrae and long bone metaphyses	Pelvis: Negative Lower limbs: positive
2	6	Pelvis	Bilateral	Patchy	Absent	none	Not perfmormed
3	3	Rachis and pelvis	Bilateral	Patchy	Present	Bilateral hip joint effusion with signal changes in the proximal femora	Not performed
4	1	Pelvis	Bilateral	Patchy	Present	Symmetrical signal changes in pelvic bones and proximal femora, with left hip joint effusion	Pelvis: Negative
5	1	Rachis, pelvis and lower limbs	Bilateral	Patchy	Present	Symmetrical signal changes in proximal femoral metaphyses and lumbar vertebrae	Pelvis: Negative Lower limbs: aspecific signs
6	1	Rachis and pelvis	Bilateral	Patchy	Present	none	Pelvis: Negative Lower limbs: aspecific signs
7	1	Pelvis and lower limbs	Bilateral	Patchy	Present	Symmetrical metaphyseal signal changes of the lower limbs with periosteal reaction and Edema of adjacent soft tissues	Pelvis: Negative
8	2	Pelvis	Bilateral	Patchy	Present	none	Pelvis: Negative
9	33	Pelvis	Bilateral	Patchy	Absent	none	Not performed
10	1	Rachis, pelvis and lower limbs	Bilateral	Homogeneous	Present	Sacroiliac joint effusion; symmetrical metaphyseal alterations of the lower limbs with periosteal reactions and soft tissue involvement	Pelvis: Negative

**Table 3:** Comparison of pelvic MRI findings in pediatric scurvy and enthesitis-related arthritis: distinguishing features between the two conditions.

PELVIC MRI FINDINGS	SCURVY	JIA/ERA
■ **Joint Effusion**	Occasional, mild	Mild to intense
■ **Joint Gadolinium Enhancement**	Mild, periosteal	Mild to intense, synovial
■ **Synovial Thickening**	Absent	Mild to intense
■ **Bone Marrow** **Changes** **Distribution**	Patchy, abnormal water-like signals (corresponding to gelatinous marrow subversion) Bilateral and symmetric	Bone marrow edema Focal and symmetric
■ **Bone Margins**	Well-demarcated	Possible erosive signs

ERA, enthesitis-related arthritis; JIA, juvenile idiopathic arthritis; MRI, magnetic resonance imaging.

## Data Availability

The datasets generated during and/or analyzed during the current study are available from the corresponding author on reasonable request. The data are not publicly available due to [The data are not publicly available due to privacy restrictions and protection of patient confidentiality.].
